# Hypothalamic Endocrine Tumors: An Update

**DOI:** 10.3390/jcm8101741

**Published:** 2019-10-20

**Authors:** Sylvia L. Asa, Ozgur Mete

**Affiliations:** 1 Department of Pathology, Case Western University and University Hospitals, Cleveland, OH 44106, USA; 2 Department of Pathology, University Health Network, Toronto, ON M5G 2C4, Canada; ozgur.mete2@uhn.ca; 3 Department of Laboratory Medicine and Pathobiology, University of Toronto, Toronto, ON M5G 1L7, Canada

**Keywords:** hypothalamus, endocrine, gangliocytoma, neurocytoma, pituicytoma, hormones

## Abstract

The hypothalamus is the site of synthesis and secretion of a number of endocrine peptides that are involved in the regulation of hormonal activity of the pituitary and other endocrine targets. Tumors of the hypothalamus have been recognized to have both structural and functional effects including hormone hypersecretion. The classification of these tumors has advanced over the last few years, and biomarkers are now available to classify these tumors and provide accurate structure–function correlations. This review provides an overview of tumors in this region that is critical to metabolic homeostasis with a focus on advances in the diagnosis of gangliocytomas, neurocytomas, and pituicytomas that are unique to this region.

## 1. Introduction

The hypothalamus is a small region of the base of the brain immediately adjacent to the pituitary gland. It is the site of synthesis and secretion of a number of neuropeptides that are involved in the regulation of hormonal activity. The axons of the neurons involved in endocrine regulation extend downwards to the median eminence, and longer axons terminate in the posterior lobe of the pituitary. Some hormones are released into the hypophysial portal vasculature where they enter the adenohypophysis and regulate the secretion of pituitary hormones. Others are secreted for transportation distally and their actions are primarily elsewhere in the body. The pituitary has often been called the conductor of the endocrine orchestra; by analogy, the hypothalamus could be considered to write the music that determines basal metabolism, growth, and reproduction, as well as appetite, temperature, and emotion. 

The anterior hypothalamus, also known as the supraoptic region, includes the supraoptic and paraventricular nuclei and several smaller nuclei that produce the major pituitary regulating hormones, corticotropin-releasing hormone (CRH), thyrotropin-releasing hormone (TRH), gonadotropin-releasing hormone (GnRH), and somatostatin (SST), as well as vasopressin (also known as anti-diuretic hormone, ADH) and oxytocin. The middle region of the hypothalamus includes the ventromedial nucleus, which is involved in the control of appetite, and the arcuate nucleus, which synthesizes growth hormone-releasing hormone (GHRH). The posterior hypothalamus, which contains the mammillary bodies, is mainly involved in temperature regulation. 

As elsewhere in the brain, the hypothalamus is composed of neurons embedded in a stroma that is composed mainly of astrocytic elements. There are vascular structures with the specific features of the blood–brain barrier, and at the periphery, there are meninges. The hypophysial portal system has its own unique properties including gomitoli that control the blood flow and retrograde flow, and there is no blood–brain barrier in the pituitary gland itself.

Tumors of this region arise from the various components described. These are summarized in Table 1. Other lesions, including hamartoma, cysts, and inflammatory lesions, will not be discussed in this review.

## 2. Clinical Manifestations of Hypothalamic Tumors

Patients with a tumor in the basal hypothalamus present with clinical signs and symptoms that fall into two general categories: Those that result from the effects of the tumor mass, and those attributable to hormone hypersecretion by the tumor.

The presence of a mass in the region immediately adjacent to the sella turcica results in increased intracranial pressure and compression on surrounding structures. Thus, patients can develop headache, nausea, and vomiting. They often develop visual field disturbances due to compression and stretching of the optic chiasm; the classical finding is a bitemporal hemianopsia, but the tumors may affect one side more than the other. Rarely, involvement and destruction of the various hypothalamic nuclei by extremely large tumors can result in changes in appetite that cause weight loss or weight gain, temperature regulation can be affected, and in some patients, sleep patterns are dysregulated. Severe alterations include blood pressure dysregulation and breathing patterns. The hypothalamus is critical in emotion control and this too can be altered, resulting in psychological changes such as anger, confusion, and depression, and altered ability to control the autonomic responses of breathing, pulse, and blood pressure to emotional stimuli. 

The hormonal impact of these tumors varies with the tumor type with the exception of its effects on the pituitary. Since the hypothalamus is responsible for the stimulation of most pituitary hormones, tumors in this region usually result in hypopituitarism, apart from prolactin, which is under tonic inhibition by hypothalamic dopamine, and therefore is increased in patients with hypothalamic tumors; the exception to this rule is if the lesion infiltrates and destroys the sella turcica. Depending on the hormonal product of the hypothalamic tumor, patients may have acromegaly due to excess GHRH [1,2,3], Cushing disease due to CRH excess [4], or even low levels of vasopressin excess [5] or significant hyperprolactinemia [6,7,8]. The expression of GnRH results in precocious puberty when they occur in children [9,10,11]. Patients with tumors producing vasopressin in significant quantities develop the syndrome of inappropriate diuresis (SIAD), which is characterized by signs and symptoms that result from volume overload and hyponatremia; these include nausea and/or vomiting, cramps, tremors, and seizures, but also more subtle features including depression, irritability, memory problems, and hallucinations.

## 3. Tumor Classification

As noted in Table 1, there are several types of tumors that occur in this region. Some are unique to the hypothalamus, as they are composed of hypothalamic neurons, but they resemble tumors composed of neurons elsewhere in the brain. Tumors of neural stroma include gliomas but also those derived from the modified special glia known as “pituicytes” of the posterior pituitary. Other lesions that occur in this area are not unique to this location, but are important in the differential diagnosis. 

### 3.1. Neuronal Tumors

***Gangliocytoma*** is a well-differentiated slowly-growing tumor composed of mature neurons that resemble normal hypothalamic neurons [12,13]. Occasional tumors may have a neoplastic glial component; these rare tumors are called “gangliogliomas” [14]. In the past, these tumors have been classified as “hypothalamic hamartoma” or “choristoma”, but these terminologies should no longer be used, especially because “hypothalamic hamartoma” is now known to represent a different entity. Many of these tumors have been associated with associated adenohypophysial pathology [12,13]. The majority have been associated with acromegaly and the production of GHRH but the other clinical manifestations of hormone excess include Cushing disease, hyperprolactinemia, and precocious puberty. There has been some controversy about whether they should be called “hypothalamic”, because they may occasionally be purely intrasellar, however, the tumor cell differentiation is clearly hypothalamic [3,12,15].

**Figure 1:**
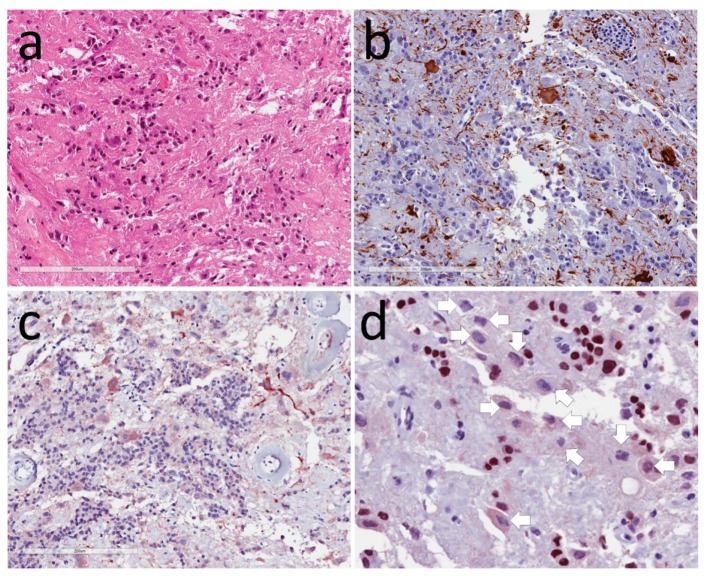
Hypothalamic gangliocytoma associated with a somatotroph tumor. (**a**)Hematoxylin and eosin (H&E) stain identifies two cell populations; (**b**) Neurofilament highlights the neurons; (**c**) Growth hormone-releasing hormone (GHRH) is localized to the neurons; (**d**) PIT1 stains the nuclei of somatotroph, but not neurons (arrows).

These tumors are composed of large mature ganglion cells that vary in size and shape with abundant cytoplasm containing Nissl substance (Figure 1); they may be binucleated or even multinucleated. By electron microscopy, they resemble hypothalamic neurons [2,3,4,16,17]. They stain for synaptophysin, MAP2, S100, and NeuN, as well as neurofilaments that highlight the axons and dendrites [5,18]. Glial elements are identified with glial fibrillary acidic protein (GFAP). Mitoses are not usually seen, and the Ki-67/MIB-1 labeling index is usually very low. Some tumors have focal calcification in their collagenous stroma, and there may be blood vessel proliferation. There is usually intermingled adenohypophysis that may be nontumorous [5], but more frequently it is also neoplastic, and may even camouflage the neuronal component; there is usually intimate association of the two cell types seen on ultrastructural examination [2,3,4,16,17]. Immunohistochemistry identifies hypothalamic hormones, including GHRH, glucagon, somatostatin, vasoactive intestinal peptide (VIP), corticotrophin-releasing hormone (CRH), gonadotropin-releasing hormone (GnRH), gastrin, encephalin, vasopressin, oxytocin, galanin, and serotonin [3,4,6,8,9,16,19,20,21,22,23,24], but some have also been reported to express adenohypophysial hormones such as prolactin [6,25] and products of pro-opiomelanocortin such as adrenocorticotropic hormone (ACTH), β-endorphin, and β-lipotropin.

The pathogenesis of these lesions is unknown, but their frequent association with adenohypophysial neoplasms suggests a common causation [1,26]. One possibility is a common stimulus [1], and this is supported by the occurrence of such a lesion in a patient with multiple endocrine neoplasia type 1 [27]. However, the other possibility is divergent differentiation or transdifferentiation [1,26,28], which is a hypothesis that has been supported by the recent report of nuclear reactivity for the pituitary transcription factor PIT1 in the ganglion cells of such a tumor [18].

***Neurocytoma*** is a rare hypothalamic tumor that is related to other central nervous system (CNS) neurocytomas which are either “central”, originating within the lateral ventricles, or “extraventricular”, arising in the cerebral hemispheres, brainstem, cerebellum, or spinal cord. These tumors composed of small neurons are usually low grade with inconspicuous mitoses and Ki67-proliferation indices below 2%; occasional tumors with necrosis, microvascular proliferation, and three or more mitoses per 10 high power fields or a Ki-67 labeling index >3% are classified as “atypical” and have been associated with a worse prognosis [14,29,30].

Neurocytomas of the hypothalamus usually present as mass lesions, and the majority have caused SIAD due to excess vasopressin secretion [5,31,32]. There is a case of acromegaly attributed to GHRH production by such a tumor [33], and some have had no features of hormone production [34,35]. It is likely that some reports of sellar or parasellar tumors classified as “olfactory neuroblastoma” (also called “esthesioneuroblastoma”) were actually hypothalamic neurocytic neoplasms [36,37,38,39,40].

**Figure 2:**
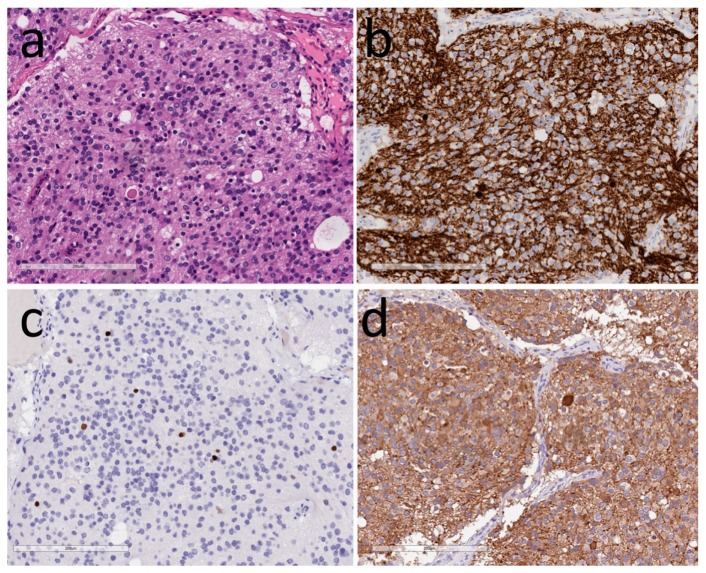
Hypothalamic neurocytoma. (**a**) H&E identifies small round cells in abundant neuropil with a dilated neuronal axon resembling a Herring body; (**b**) Neurofilament highlights the neurons and neuropil; (**c**) TTF1 decorates some of the neurons; (**d**) Vasopressin is expressed by the tumor cells and highlights an axonal terminal known as a Herring body.

The histologic appearance is characterized by monotonous sheets and nests of small to medium-sized round cells that form occasional rosette-like structures within a vascular fibrillary neuropil (Figure 2). The tumor cell nuclei are round to oval with finely granular chromatin and multiple nucleoli; their cytoplasm is pale eosinophilic to chromophobic and granular. Occasional acidophilic hyaline globules within the neuropil resemble Herring bodies of the posterior pituitary. There may be fibrosis and calcification. Mitoses and microvascular proliferation are rare and indicate a more aggressive tumor [30]. Electron microscopy shows polygonal tumor cells with multiple elongated neuritic processes with microtubules and dense core secretory granules associated with synaptic junctions [31,32]. Immunohistochemistry localizes synaptophysin, chromogranin-A, and neurofilaments in the cytoplasm, as well as variable NeuN and Thyroid Transcription Factor 1 (TTF1) in the nuclei [41,42]; S100 protein, calretinin, and CD99 may be expressed. Vasopressin has been shown in tumors causing SIAD, and GRH was reported in a case of acromegaly. 

The differential diagnosis of these tumors includes adenohypophysial neuroendocrine tumors, paraganglioma, and olfactory neuroblastoma. These tumors are negative for keratins, pituitary transcription factors, and hormones, as well as tyrosine hydroxylase, allowing the distinction of the first two, and positivity for TTF1 and hypothalamic hormones allows the recognition of these as hypothalamic rather than olfactory in differentiation and origin.

The pathogenesis of sellar neurocytomas is not known, but some information can be gleaned from molecular studies of extraventricular neurocytomas. Those tumors do not harbor alterations seen in other brain tumors such as methylation of the promoter of O6-methylguanine-DNA methyltransferase (*MGMT*), co-deletion of 1p/19q, or mutations of isocitrate dehydrogenase enzyme isoform 1 (*IDH1*), *IDH2, alpha-internexin*, or *Tp53* [30]. Two tumors examined by array-based comparative genomic hybridization showed different profiles with the loss and gain of multiple chromosomal loci [30]. There is a single case report of polysomy of the epidermal growth factor receptor (*EGFR*) gene [30].

### 3.2. Tumors of Glia

***Pituicytoma*** is a tumor of the specialized modified glial cells of the posterior pituitary. These cells are known as “pituicytes”, hence the name. This tumor has been recognized as “pituicytoma” when it takes the classical form of a spindle cell tumor, but there are several variants that were initially misclassified; these include “spindle cell oncocytoma; formerly known as spindle cell oncocytoma of the adenohypohysis” and “granular cell tumor” as well as “sellar ependymoma”. A careful study of the normal posterior pituitary has shown that there are several variants of pituicytes including oncocytic, granular cell, and ependymal types [43,44] that explain these variants of pituicytomas [45]. Similar to normal pituicytes that derive from the basal hypothalamus and express TTF1 (clone SPT24), these tumors all have as their hallmark expression of the TTF1 transcription factor [44,45,46]. 

Pituicytoma is a hormonally inactive intrasellar or suprasellar mass that usually presents with headache, visual disturbance, and hypopituitarism [47,48,49,50,51]. The granular cells variant tends to be slower growing, and is often found as a small, clinically asymptomatic incidental finding; many are seen only at autopsy [52,53]. Despite their location in the posterior lobe, only rarely are these tumors associated with diabetes insipidus [54], but this is explained by the fact that the cell bodies of the vasopressin-secreting neurons remain intact in the hypothalamus. 

**Figure 3:**
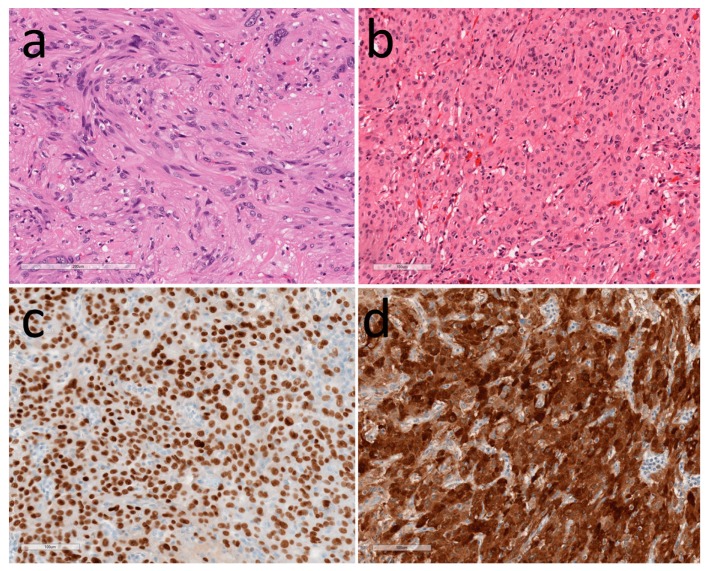
Pituicytomas. (**a**) H&E of a classical pituicytoma shows spindle-shaped cells forming fascicles; (**b**) An oncocytic variant that is composed of round cells with granular eosinophilic cytoplasm; (**c**) TTF1 decorates the nuclei of the tumor cells; (**d**) S100 is expressed by the tumor cells.

Pituicytomas are characterized by elongated eosinophilic spindle-shaped cells forming interlacing fascicles known as a “storiform pattern” (Figure 3). The tumor cells have distinct cell borders and minimal nuclear atypia. The oncocytic variant (spindle cell oncocytoma) is composed of plump epithelioid cells with more abundant eosinophilic granular cytoplasm. The granular cell variant has polygonal cells with conspicuous granular eosinophilic cytoplasm, and the ependymal variant forms ependymal-type rosettes. By electron microscopy, the tumor cells are spindled or polygonal with well-formed desmosomes and intercellular junctions, but there are no secretory granules; oncocytic variants have abundant dilated mitochondria, whereas granular cell variants have phagolysosomes with electron-dense membranous debris [55].

Immunohistochemistry localizes the S100 protein, vimentin, and GFAP that may be focal or weak; epithelial membrane antigen (EMA) is usually positive, but may be negative [56]. Pituicytomas are negative for synaptophysin, chromogranin, neurofilaments, and keratins, as well as adenohypophysial transcription factors and hormones, CD34, bcl-2, smooth muscle actin and desmin. They consistently stain for TTF1 [44,45,46]. They also express galectin-3 [47,57], but this is not a distinguishing feature. The oncocytic variant stains avidly for mitochondrial antigens. Granular cell tumors show reactivity for biomarkers of lysozymes including CD68, alpha-1-antitrypsin, alpha-1-antichymotrypsin, and cathepsin B [58], as well as strong positivity with the periodic acid Schiff (PAS) stain.

***Gliomas*** that arise in the hypothalamus, in the nearby optic pathway, or in the region of the inferior third ventricle are rare [59,60]. They can mimic adenohypophysial neuroendocrine tumors, presenting as a parasellar and sellar mass. The most aggressive gliomas in this location have been reported as sequelae of radiation therapy for primary pituitary tumors [61,62,63,64,65,66,67,68,69,70,71]. The most common glioma in this region is the pilocytic astrocytoma, also known as “optic glioma”, which is more common in children and adolescents [72,73,74] and may be associated with neurofibromatosis type I (NF1) [75,76]. The biologically more aggressive variant is classified as pilomyxoid astrocytoma, which is most common in infants [77,78].

### 3.3. Other Tumors in the Hypothalamus

Many other tumors can occur in and around the hypothalamus [79]. Some are infiltrating primary pituitary tumors, most commonly pituitary neuroendocrine tumors (PitNETs) [80], but also craniopharyngiomas. Teratomas and germ cell tumors occur in the midline and can involve the hypothalamus. Occasionally, schwannomas occur associated with nerves in the parasellar region, and meningiomas have been seen in the hypothalamic dura. Chordomas may arise in the clivus, and vascular and mesenchymal neoplasms of many types have been reported. The one unique vascular lesion in this region is the glomangioma, which is thought to arise from the gomitoli of the hypothalamic–pituitary portal vasculature [81,82]. Lymphomas are rare in this location, but it is a site of involvement by histiocytoses. 

## 4. Implications of Pathology Diagnosis

The importance of pathology in the classification of hypothalamic tumors is evident, given the vast array of lesions that can occur in this region, which is critical for homeostasis. This review has focused only on neoplasms, but other tumor-like lesions occur as well, including cysts and inflammatory lesions. The importance of clinicopathologic correlations cannot be overemphasized, especially for tumors that are hormonally active, and the application of appropriate biomarkers plays a critical role in determining the accurate pathology diagnosis and therapeutic plan for every patient with a hypothalamic lesion. 

## Figures and Tables

**Table 1:** Tumor Classification.

Neuronal Neoplasms	Gangliocytomas
	Neurocytomas
Glial Neoplasms	Gliomas
	Pituicytomas (including oncocytic, ependymal, and granular cell variants) Hypothalamic and optic gliomas
Neural Stromal Neoplasms	Schwannomas
	Meningiomas
	Chordomas
Other Stromal Neoplasms	Vascular and mesenchymal tumors
	Lymphomas
	Germ cell tumors
Infiltrating Neoplasms	PitNETs (Pituitary neuroendocrine tumors)
	Craniopharyngiomas
	Germ cell tumors, including teratomas
Metastatic Neoplasms	
